# Spatial Patterns in Herbivory on a Coral Reef Are Influenced by Structural Complexity but Not by Algal Traits

**DOI:** 10.1371/journal.pone.0017115

**Published:** 2011-02-11

**Authors:** Adriana Vergés, Mathew A. Vanderklift, Christopher Doropoulos, Glenn A. Hyndes

**Affiliations:** 1 Sydney Institute of Marine Science and Evolution and Ecology Research Centre, School of Biological, Earth and Environmental Sciences, University of New South Wales, Sydney, Australia; 2 Centre for Marine Ecosystems Research, School of Natural Sciences, Edith Cowan University, Joondalup, Australia; 3 CSIRO Wealth from Oceans Flagship, Wembley, Australia; 4 School of Biological Sciences and ARC Centre of Excellence for Coral Reef Studies, The University of Queensland, Brisbane, Australia; University of Canterbury, New Zealand

## Abstract

**Background:**

Patterns of herbivory can alter the spatial structure of ecosystems, with important consequences for ecosystem functions and biodiversity. While the factors that drive spatial patterns in herbivory in terrestrial systems are well established, comparatively less is known about what influences the distribution of herbivory in coral reefs.

**Methodology and Principal Findings:**

We quantified spatial patterns of macroalgal consumption in a cross-section of Ningaloo Reef (Western Australia). We used a combination of descriptive and experimental approaches to assess the influence of multiple macroalgal traits and structural complexity in establishing the observed spatial patterns in macroalgal herbivory, and to identify potential feedback mechanisms between herbivory and macroalgal nutritional quality. Spatial patterns in macroalgal consumption were best explained by differences in structural complexity among habitats. The biomass of herbivorous fish, and rates of herbivory were always greater in the structurally-complex coral-dominated outer reef and reef flat habitats, which were also characterised by high biomass of herbivorous fish, low cover and biomass of macroalgae and the presence of unpalatable algae species. Macroalgal consumption decreased to undetectable levels within 75 m of structurally-complex reef habitat, and algae were most abundant in the structurally-simple lagoon habitats, which were also characterised by the presence of the most palatable algae species. In contrast to terrestrial ecosystems, herbivory patterns were not influenced by the distribution, productivity or nutritional quality of resources (macroalgae), and we found no evidence of a positive feedback between macroalgal consumption and the nitrogen content of algae.

**Significance:**

This study highlights the importance of seascape-scale patterns in structural complexity in determining spatial patterns of macroalgal consumption by fish. Given the importance of herbivory in maintaining the ability of coral reefs to reorganise and retain ecosystem functions following disturbance, structural complexity emerges as a critical feature that is essential for the healthy functioning of these ecosystems.

## Introduction

Spatial heterogeneity in ecosystems can strongly influence population structure, community composition and ecosystem processes [1]. Herbivory can generate spatial heterogeneity by regulating rates of primary production and nutrient cycling [2], [3], modifying plant community composition, diversity and biomass [4], [5], and/or directly disturbing habitats physically, e.g. through burrowing [6], [7]. Several factors are known to control the spatial distribution of herbivory, including abiotic influences such as topography or distance to water (in terrestrial systems), and biotic influences such as plant distribution, nutritional quality, predation, herbivore social behaviour (e.g. herding), and human management practices [8], [9], [10], [11]. Additionally, feedback mechanisms between herbivory and plant quality can also influence spatial patterns of herbivory. For example, while herbivory generally decreases plant biomass, it often enhances nutrient recycling and availability [12], [13], although these short-term positive feedbacks may eventually result in a compositional shift towards less palatable plant species [14]. However, much of this knowledge comes from wildlife and rangeland management literature that deals mostly with large ungulates, and we know comparatively less about what controls spatial patterns of herbivory in ecosystems characterised by other consumers.

This study focuses on the processes that control the spatial distribution of herbivory in coral reefs, ecosystems that are characterised by some of the highest rates of herbivory [3], [15], [16]. Herbivores can remove over 90% of daily algal production in shallow coral reefs [3], [17], [18], and the presence of abundant coral depends on high levels of herbivory [19], [20], [21]. Indeed, herbivorous fish play a crucial role in maintaining coral-reef resilience (i.e. the ability of a system to absorb disturbance whilst maintaining ecosystem function [22]) by consuming macroalgae that can otherwise outcompete corals when new space becomes available following disturbance [23], [24], [25], [26], [27]. However, the impact of herbivores is usually not uniform across all habitats, and coral reefs may be viewed as spatial mosaics of animal- and macroalgae-dominated communities characterised by different intensities of herbivory [19], [28], [29], [30]. Thus, variations in the intensity of herbivory between different parts of a reef separated by short distances (tens to hundreds of metres) can be greater than differences among reefs that are many kilometres apart [31]. Despite the existence of such marked spatial patterns in herbivory in coral reefs, we have a limited knowledge of the factors that drive differences across reef gradients.

Early studies dealing with spatial patterns in coral reefs focused on the distribution of marine plants, and highlighted the importance of herbivory for maintaining differences among habitats [17], [19], [28], [32], [33]. Other studies focused on the distribution of herbivores in different sections of the reef and found marked variations in densities and species composition [29], [30], [31], [34]. Several mechanisms involving both abiotic and biotic influences have been proposed to explain the striking gradients in the intensity of herbivory observed on coral reefs worldwide. Among the abiotic influences, wave exposure and depth are considered to inhibit herbivory. Generally, herbivory is often lowest in the first 1–2 meters of water because turbulence associated with wave impact hinders the feeding ability of fish, and it is usually greatest a few meters below the surface and decreases thereafter at depths greater than 20 m [35], [36], [37]. In contrast, structural complexity and availability of refuges are considered to enhance herbivory [29], [32], [33], [35]. In terms of biotic factors, large grazers appear to aggregate in zones of high algal turf production, although the mechanisms by which fish respond to productivity are not clear [38]. Despite many such hypotheses having been invoked to explain spatial variation of herbivory in coral reefs, few studies have experimentally tested the importance of specific mechanisms. Moreover, herbivory in coral reefs is a process that involves a wide range of consumers with highly variable feeding modes and diets and with contrasting ecological functions [39], and there is a need to quantify and assess the impact of different functional groups separately. For example, differences in turf algae productivity may influence (and be influenced by) the distribution of fishes that consume turf algae [38], but probably have no effect on species that feed on macroalgae.

Roving herbivorous fishes have been clearly identified as the key herbivores in undisturbed coral reefs [21]. However, they do not constitute an ecologically uniform group, but can be broadly classified into grazer and browser functional groups, depending on their diet and mode of feeding [40], [41]. Grazing taxa (including scraping and excavating parrotfishes) typically feed on the epilithic algal matrix (EAM sensu Wilson [42]) and crustose coralline algae, and constitute the majority of herbivorous fishes on coral reefs. In contrast, only a handful of species are considered to be browsers – that is, species that consume large erect macroalgae [43], [44], [45], [46]. Grazers and browsers are thought to play distinct and complementary roles in avoiding phase shifts towards macroalgal dominance [40], [47]. Grazers can preclude an increase in overall algal biomass, prevent macroalgal growth by consuming macroalgal recruits, and provide space for coral recruitment, while browsers consume the adult brown seaweeds that typically dominate coral reefs in the absence of herbivory, and therefore have the potential to reverse phase shifts once macroalgae are established in reefs [40], [47]. Recent studies have highlighted the importance of macroalgal consumption and have identified the key species or functional groups responsible for this ecological function [30], [45], [46], [47], [48], but we know little about the mechanisms that control the distribution and abundance of these browsers.

In this study, we quantified spatial patterns of macroalgal herbivory by fishes across a coral reef, tested for similar spatial patterns in potential explanatory variables, and then used manipulative and mensurative experiments to test some hypotheses arising from the patterns observed. We tested for the presence of spatial variation in herbivory by quantifying consumption of erect macroalgae and measuring the biomass and composition of herbivorous fishes among a cross section of a coral reef (lagoon, reef flat and outer reef habitats). We then related patterns in herbivory to the spatial distribution of algal cover, algal biomass and structural complexity in these three habitats. In the manipulative and mensurative experiments, we selected two habitats with contrasting levels of herbivory (reef flat and lagoon) and used herbivore exclusion and feeding experiments to test hypotheses about the mechanisms that might cause the observed consumption patterns. In particular, we asked: (1) Does consumption of macroalgae relate to spatial patterns in macroalgal productivity, nutritional quality, community composition and/or palatability? (2) Does herbivory influence macroalgal nutritional quality? (3) Does benthic structural complexity and proximity to reef influence the distribution of macroalgal herbivory?

## Results

### Seascape patterns in the distribution of herbivory, herbivores, macroalgae, coral cover and rugosity

**Figure 1:**
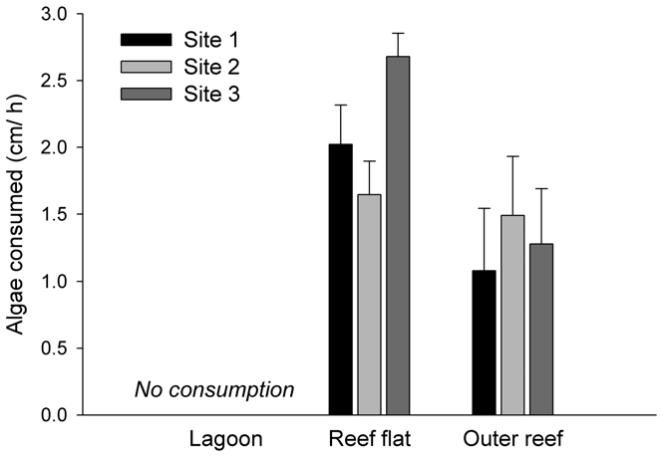
Seascape patterns in the distribution of herbivory. Length of *Sargassum myriocystum* lateral branches consumed per hour (mean ± SE) at lagoon, reef flat and outer reef habitats at each of the experimental sites.

There was a significant difference in the rates of herbivory (measured as consumption of tethered *Sargassum*) among habitats, a pattern that was constant at all sites (Fig. 1, Table S1). No consumption was recorded in the lagoon, whereas in the reef flat and outer reef habitats we found similar rates of about 1–2 cm h^−1^ (permutational analysis of variance [PERMANOVA] pair-wise tests: Lagoon < Reef flat  =  Outer reef; p<0.02). Since *Sargassum* was much more abundant in the lagoon than in other habitats (see below), and consumption may depend on local availability of this resource, we incorporated *Sargassum* availability into our analysis of consumption by comparing a relative consumption index (RCI) for this taxa across habitats, where RCI  =  proportion of *Sargassum* consumed x proportion of *Sargassum* present (from algae biomass results, data averaged at site level; method modified from the ‘global preference index’ used by Hoey and Bellwood [45]). We found equally striking differences between habitats in RCI (F_2, 6_ = 15.89; p = 0.004; data not shown) as for rates of herbivory.

**Figure 2:**
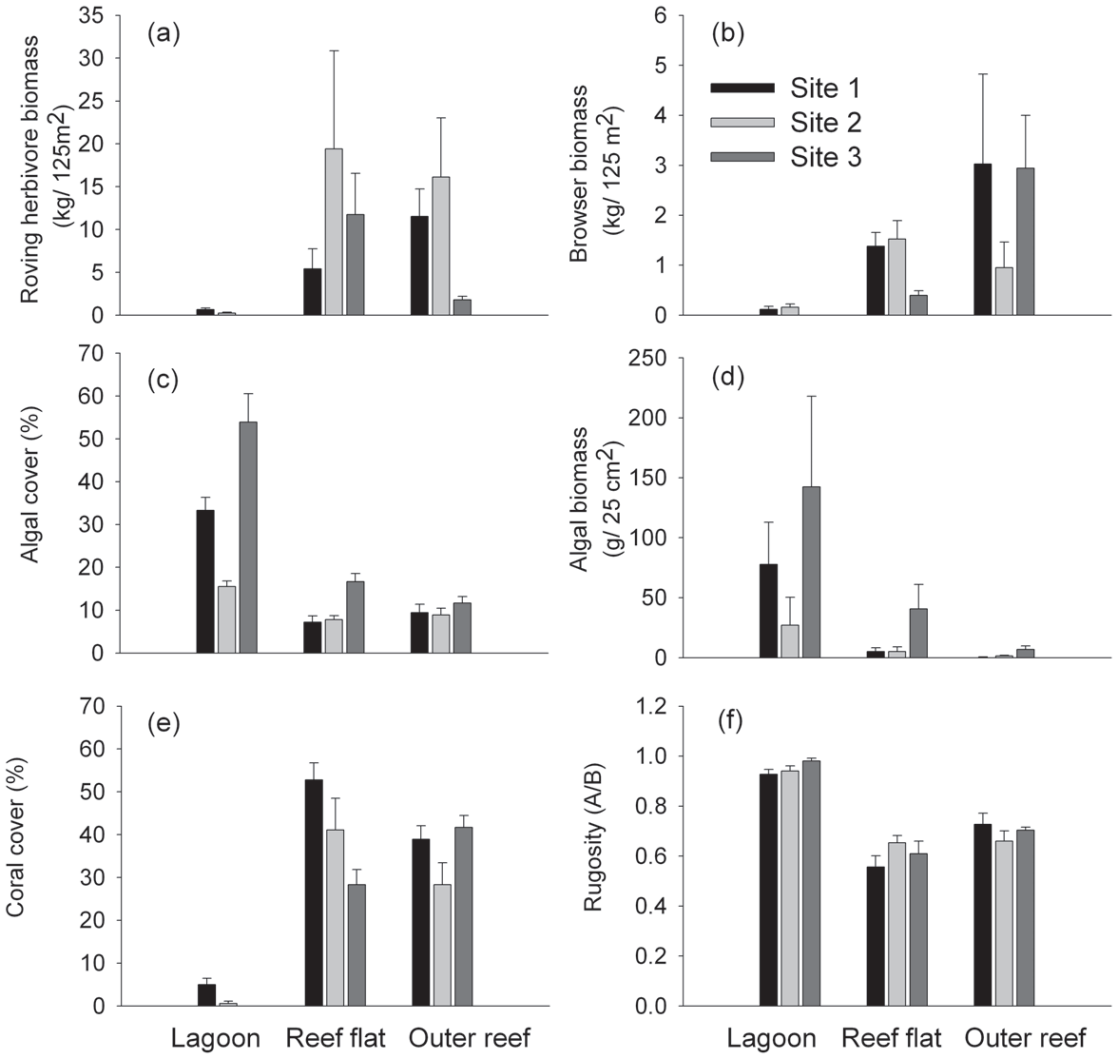
Seascape patterns in the distribution of herbivores, macroalgae, coral cover and rugosity. Data represent means ± SE of (a) total roving herbivorous fish biomass, (b) total browsing fish biomass, (c) algal cover, (d) algal biomass, (e) coral cover, and (f) rugosity.

We found strong differences among habitats in total biomass of all roving herbivorous fish and of browsing fish alone, a pattern that was consistent at all sites (Table S2a, S2b). The reef flat and outer reef habitats generally hosted an order of magnitude higher herbivore biomass than the lagoon (Fig. 2a, 2b; PERMANOVA pair-wise tests, p<0.05 for comparison between lagoon and either outer reef or reef flat). The species composition of roving herbivorous fish assemblages was different among habitats but the nature of these differences varied among sites (significant Habitat x Site interaction; Table S3a). Pair-wise comparisons showed that roving herbivorous fish assemblages in the three habitats were significantly different from each other at all sites (p≤0.002 for all comparisons), but within each habitat, assemblages were only similar among sites in the reef flat and outer reef habitats, and not in the lagoon – i.e. the interaction was caused by the greater degree of variability in the lagoon. Differences in the composition of the browser fish assemblages between habitats were less consistent across sites (significant Habitat x Site interaction yielded by the PERMANOVA analysis: Table S3b), with significant differences among all three habitats at two of the sites (PERMANOVA pair-wise tests, p<0.05 for all comparisons), but not at the other site, where only lagoon and reef flat assemblages differed (PERMANOVA pair-wise test, p = 0.008).

**Figure 3:**
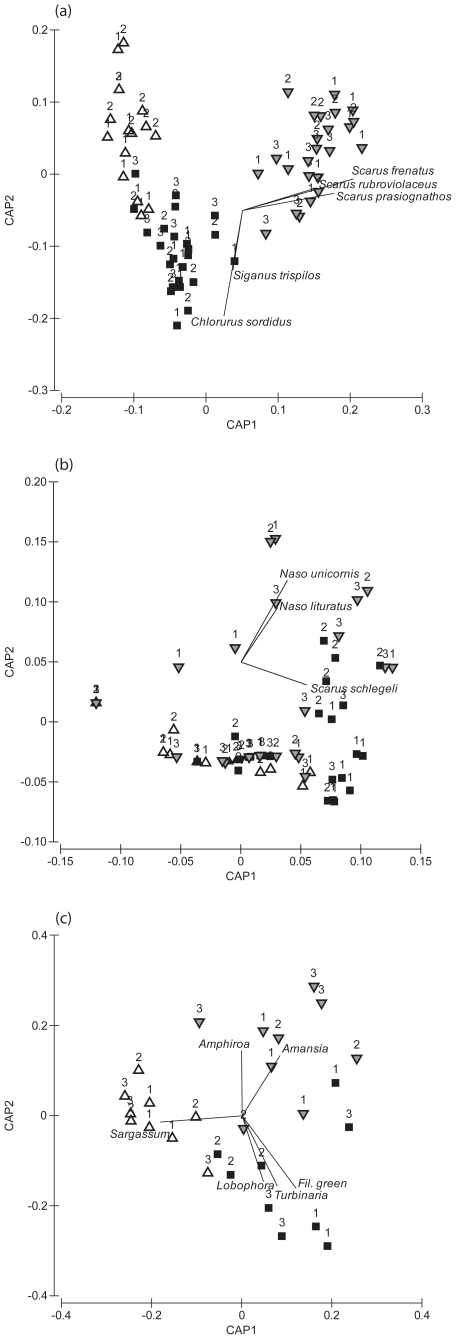
Seascape patterns in the distribution of fish and algae assemblages. Canonical analysis of principal coordinates (CAP) comparing community assemblages of (a) all roving herbivorous fish, (b) all browsing fish, and (c) macroalgae between sites (numbered icons) and habitats (symbols): Triangles facing upwards  =  Lagoon; Triangles facing downwards  =  Outer reef; Squares  =  Reef flat habitat. Data were fourth-root transformed prior to ordination.

Canonical analysis of principal components (CAP) of all roving herbivorous fish yielded a high classification success of 93.1% across all habitat types (i.e. only 6.9% misclassification error). Correlations with CAP axis scores indicate that a high biomass of *Chlorurus sordidus* was characteristic of the reef flat (Fig. 3a), where the biomass of this species was about an order of magnitude higher than that in the lagoon or outer reef. Although less abundant, *Siganus trispilos* was also characteristic of the reef flat habitat. *Scarus frenatus*, *S. prasiognathos* and *S. rubroviolaceous* characterised the outer reef habitat, with average biomass for each species in the outer reef 20 times higher than those in the other habitats. No species were identified as characteristic of the lagoon habitat; this habitat was instead characterised by a low biomass of all species. CAP of browser fish assemblages yielded a low classification success of 59.7% (Fig. 3b). Correlations with CAP axis scores indicated that the outer reef tended to be characterised by high biomasses of *Naso unicornis* and *N. lituratus*, while the reef flat was characterised by higher biomass of *Scarus schlegeli* (Fig. 3b).

There were differences among habitats and sites in algal cover (Fig. 2c; Table S2c) and among habitats in algal biomass (Fig. 2d; Table S2d). Algal cover and biomass were higher in the lagoon than in either the reef flat or the outer reef, which were similar (SNK pair-wise comparisons: Lagoon > Reef flat  =  Outer reef, p<0.05). Differences in macroalgal species assemblages among habitats were not consistent among sites (significant Habitat x Site interaction; Table S3c). Lagoon and outer reef habitats differed significantly at all three sites (p<0.05), but comparisons of reef flat habitat with lagoon and outer reef were not significant anywhere. CAP yielded a classification success of 70.4% across all habitat types (i.e. 29.6% misclassification error). The reef flat habitat hosted high biomass of *Lobophora variegata*, *Turbinaria ornata* and an unidentified filamentous green alga, whereas the outer reef habitat was characterised by red algae belonging to the genera *Amphiroa* and *Amansia* (Fig. 3c). The lagoon habitat was strongly characterised by *Sargassum* species, which represented over 80% of the total algal biomass in this habitat.

Coral cover differed among habitats, but the nature of this difference varied among sites (significant Site x Habitat interaction; Fig. 2e, Table S2e). Coral cover was always lower in the lagoon than in any other habitat (0–5% overall cover; p≤0.002 for all comparisons), but differences in coral cover between reef flat and outer reef were not consistent between sites. There were clear differences in rugosity among habitats, a pattern that was consistent at all sites (Fig. 2f, Table S2f). The lagoon was the least structurally complex habitat, with rugosity values approaching 1 (mean all sites 0.95±0.01; p≤0.01 for all comparisons), whereas the outer reef had similar rugosity values to the reef flat, which were ∼50% more structurally complex than the lagoon (Fig. 2f).

### Relationships between macroalgal herbivory and other variables

**Figure 4:**
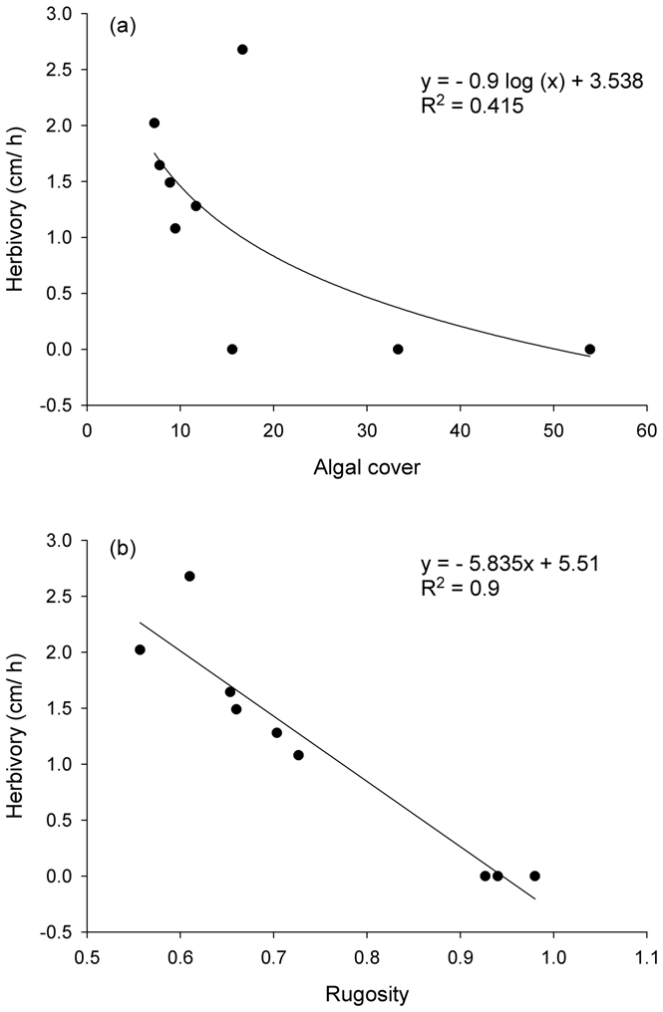
Relationships between rates of herbivory, algae cover and rugosity. (a) Logarithmic relationship between herbivory rates and algal cover. (b) Linear relationship between herbivory rates and rugosity. All variables were averaged for each site.

We found a near-significant logarithmic relationship between site averages for measurements of macroalgal herbivory and algal cover (Fig. 4a; F_1, 7_ = 4.958; p = 0.06; r^2^ = 0.415) but not between macroalgal herbivory and algal biomass (F_1, 7_ = 3.26; r^2^ = 0.318; p = 0.11). In addition, there were no significant relationships between measurements of herbivory rates and total herbivorous fish biomass (F_1, 7_ = 1.392; r^2^ = 0.166; p = 0.277), or browser fish biomass (F_1, 7_ = 0.822; r^2^ = 0.105; p = 0.395). Rates of herbivory were higher in the sites and habitats that were more structurally complex, as reflected by a strong linear relationship between rates of consumption of tethered *Sargassum* and rugosity (Fig. 4b; F_1, 7_ = 64.82; r^2^ = 0.90; p<0.001), where rugosity explained 90% of the variance in consumption.

### Experimental test of effects of habitat and herbivory on algal consumption, productivity and chemical composition

**Figure 5:**
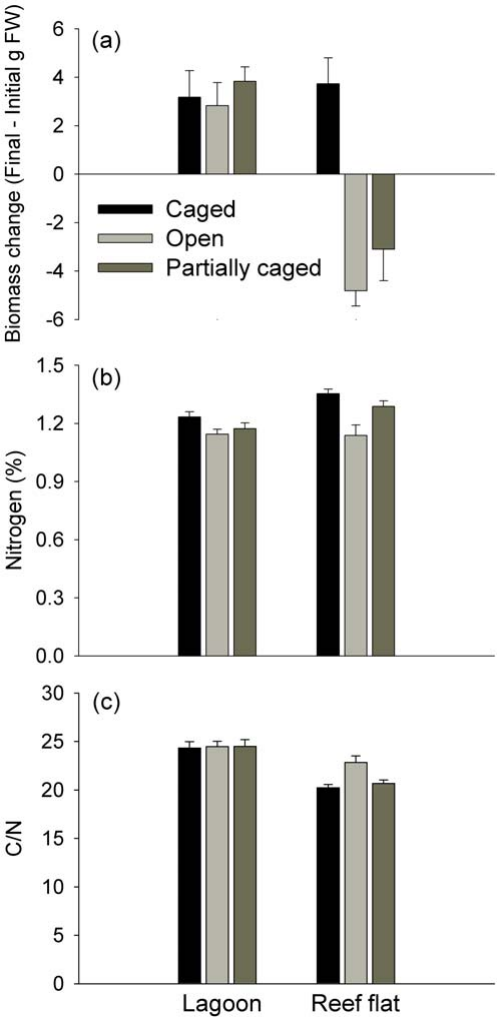
Experimental test of effects of habitat and herbivory on algal consumption, productivity and chemical composition. (a) Biomass change and (b) nitrogen and (c) carbon/nitrogen ratio of *Lobophora variegata* transplanted to reef flat and lagoon habitats in three experimental treatments designed to manipulate access by herbivores (Caged, Open and Partially caged) after 6 weeks. Data pooled across the three sites, bars represent means ± SE.

Herbivore exclusion experiments performed with *Lobophora variegata* in high and low herbivory habitats (reef flat and lagoon, respectively) showed that changes in algae biomass were strongly influenced by the habitat in which thalli were deployed and whether thalli were caged, as shown by a Habitat x Herbivory interaction (Fig. 5a, Table S4a). In the reef flat habitat, caging had an acute effect on biomass change: there was a net increase in biomass of 30% inside cages, compared to a net decrease in biomass of 30–50% in the partial and open cages (SNK pairwise comparisons: Caged > Open  =  Partially Caged; p<0.01). In contrast, caging had no effect on algal biomass in the lagoon habitat, where there was a net increase in biomass of 30–60% in all treatments (SNK pairwise comparisons: Caged  =  Open  =  Partially Caged). The clear inference from this result is that herbivores strongly reduced *L. variegata* biomass on the reef flat, but not in the lagoon. No artefacts were associated with the structure of the cages (SNK pairwise comparisons: Partially Caged Lagoon  =  Open Lagoon, and Partially Caged Reef flat  =  Open Reef flat), i.e. the presence of cages did not confound the interpretation of the effects of herbivory on algal biomass. In the absence of herbivory, there were no differences in *L. variegata* biomass accumulation between habitats (Fig. 5a; SNK pairwise comparisons not significant: Reef flat Caged  =  Lagoon Caged).

Nitrogen content was significantly higher in caged algae than in open and partial cages (PERMANOVA pair-wise tests p<0.05) (Fig. 5b, Table S4b), but the habitat in which algae were placed had no effect on nitrogen content. In contrast, the availability of nitrogen per unit carbon (C:N ratio) was not affected by caging but was significantly influenced by the habitat in which algae were placed, with highest C:N ratios found in algae transplanted to lagoon habitats (Figure 5c, Table S4c).

### Experimental test of palatability of algae from high and low herbivory habitats

We detected no significant difference in area loss between lagoon and reef flat morphotypes of *Lobophora variegata* after 5 days of deployment (mean consumption (± SE) reef flat morphotype  = 2.57±1.14 cm^2^, lagoon morphotype  = 2.13±0.59 cm^2^; t = −0.6359, df = 12, p = 0.537, 95% CI  =  −4.006, 1.416). The two morphotypes did not differ in their nitrogen content (mean nitrogen content (± SE) reef flat morphotype  = 1.22±0.05%, lagoon morphotype  = 1.21±0.02%; Welch's t = 0.170, df = 4.657, p = 0.873; 95% CI  =  −0.134, 0.153). The C:N ratio of the reef flat morphotype tended to be lower than the lagoon morphotype, although statistical differences between the two only approached significance (mean carbon:nitrogen ratio (± SE) reef flat morphotype  = 21.87±0.3, lagoon morphotype  = 23.65±0.81; t = −2.057, df = 8, p = 0.074). The 95% confidence intervals of this near-significant result were relatively wide and non-symmetrical around zero (95% CI = −3.757, 0.214), suggesting that a difference in C:N ratio may exist but was not detected by our test. We found no differences among morphotypes in their phenolic content (mean phenolic content (± SE) reef flat morphotype  = 1.56±0.1%, lagoon morphotype  = 1.60±0.24%; t = −0.151, df = 8, p = 0.883; 95% CI = −0.643, 0.564).

### Effect of proximity to reef on consumption of macroalgae

**Figure 6:**
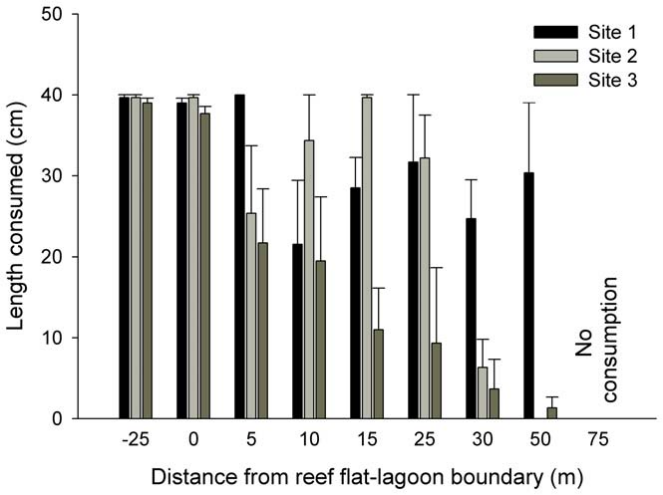
Effect of proximity to reef on consumption of macroalgae. Length of *Sargassum myriocystum* lateral branches consumed after 48 hours (mean ± SE) at increasing distances from the reef flat/lagoon boundary in the three experimental sites.

Consumption of tethered *Sargassum* varied with increasing distance from the boundary between the reef flat and lagoon, and the nature of the differences among distances varied among sites (significant Site x Distance interaction; Fig. 6, Table S5). Nearly 100% of the tethered algae offered in the middle of the reef flat (−25 m) and at the reef flat/lagoon boundary (0 m) were consumed at all sites (all SNK comparisons between sites not significant). We found variable differences in consumption among sites at distances between 5 and 50 m from the reef flat/lagoon boundary, but at all sites there was no consumption at 75 m (SNK comparisons between sites not significant). At two sites, there was either very low or no consumption at 30 and 50 m from the reef flat/lagoon boundary, but at the third site there was still high levels of consumption at those distances (significant SNK comparisons between site 1 and sites 2 and 3 at 30 m and 50 m).

## Discussion

Herbivores operate in dynamic systems where they can both generate spatial heterogeneity and respond to existing patterns in space. In this study, we found marked spatial variation in the abundance, composition and consumption of macroalgae across a coral-reef seascape. Spatial patterns in macroalgal consumption were best explained by differences in structural complexity among habitats: herbivory was always highest in the most structurally complex coral-dominated reef flat and outer reef habitats. In contrast, the cover and biomass of macroalgae appeared to be themselves influenced by consumption patterns, with habitats supporting high biomass of herbivores also supporting low algal abundance. Experimental exclusion of herbivorous fish in different habitats supported the conclusion that these consumers exert a strong influence on macroalgae in the structurally-complex reef flat habitat, but not in the structurally-simple lagoon habitat. In addition, algal consumption decreased to undetectable levels within 75 m of coral structure into the structurally simple lagoon habitat, where highly palatable macroalgae were abundant. Although productivity and nutritional quality of plants can both influence and be influenced by herbivory in terrestrial systems [12], [13], [14], we found no evidence that these traits affect the distribution of herbivory in the coral-reef seascape at Ningaloo Reef.

### Seascape patterns in the distribution of macroalgae, herbivores and herbivory

The pattern of among-habitat differences in macroalgae cover/biomass and in the composition of roving herbivorous fish in Ningaloo Reef across distances of hundreds of metres is remarkably similar to patterns observed across tens of kilometres in coral reefs with different geomorphology, such as the Great Barrier Reef (GBR). Macroalgal cover in the lagoon at Ningaloo ranged between 10–80%, values that are similar to inner shelf systems in the GBR (36–66%), while the outer reef at Ningaloo (located about 1 km offshore) hosted <10% algal cover, values that are more similar to mid-shelf or outer-shelf reefs located 50–100 km offshore in the GBR (0–15%) [34], [41]. This pattern is also apparent when comparing herbivorous fish biomass, which ranged from <1 kg/125 m^2^ in the lagoon to up to 20 kg/125 m^2^ in the reef flat and outer reef, a difference that is in the same order of magnitude as the disparity in roving herbivorous fish biomass among inner-shelf and mid/outer-shelf reefs in the GBR [34], [41].

The distinct spatial patterns in consumption of algae described in this study are also similar to the GBR [30], [33], as well as to reefs found in the Caribbean [29], [35], [49], and in the Hawaiian Islands [50]. Herbivory is always highest in coral-dominated habitats near or at the reef crest, and decreases with either depth or distance towards the inner sections of lagoons. This suggests that a similar process (or combination of processes) may be controlling the distribution of herbivory in different coral-reef ecosystems, despite great variations in their geomorphology and physical influences.

### Relationship between patterns in herbivory and macroalgal distribution, productivity, nutritional quality and palatability

We found a near-significant negative logarithmic relationship between algal cover and algal consumption that suggests that the cover of macroalgae is reduced under high herbivory conditions, but it also depends on other factors under low herbivory conditions. The conclusion that this relationship is causal, rather than just correlative, is supported by the growth in macroalgae with experimental exclusion of herbivores. We did not detect a similar relationship between macroalgal biomass and herbivory, probably because much of the algal biomass collected in our quadrats was present under coral plates and in small crevices that were effectively inaccessible to consumers, whereas algae surveyed as percentage cover better reflect algae that is available to herbivores.

Spatial patterns of macroalgal consumption were not related to among-habitat differences in macroalgal production (measured as biomass change) or nutrient content, since these algal traits were similar in habitats with contrasting levels of herbivory. Experimental exclusion of herbivores resulted in very similar growth of *Lobophora variegata* transplanted to the high herbivory reef flat and low herbivory lagoon. Similarly, we did not find any among-habitat differences in the nitrogen content of algae. We did, however, detect some differences between habitats in the availability of nitrogen per unit carbon, which tended to be higher for thalli placed in the reef flat (i.e. lower C:N ratio). Lower C:N ratios are generally associated with higher palatability, and many marine herbivores are known to preferentially consume and grow faster on diets with low C:N ratios [51], [52]. However, in our feeding preference experiment between reef flat-decumbent and lagoon-ruffled *L. variegata*, the two morphotypes were equally consumed, despite near-significant differences in C:N ratio. This contrasts with the findings of Coen and Tanner [53], who found striking differences in susceptibility to herbivory between the same two morphotypes in the Caribbean and suggested that their different morphologies were related to differential grazing intensities in their respective habitats. However, these authors also found greater chemical differences among morphotypes than detected in our study, which may explain the discrepancy with our results.

Differences in the algal assemblages from habitats with contrasting levels of herbivory are consistent with the inference that macroalgal consumers are influencing spatial patterns in macroalgal community composition. The habitat with the lowest rates of herbivory (lagoon) was characterised by high abundance of palatable *Sargassum* species, which are readily eaten by macroalgal browsers worldwide when accessible (pers. obs.; [45], [54], [55], [56] and are actively selected by siganids [57]. Of the algae that characterised reef flat habitats, *Turbinaria ornata* is considered unpalatable [58], while *Lobophora variegata* is consumed by browsers to varying degrees (pers. obs.; [54], [59], [60], [61], [62]. Outer reef habitats with similar levels of herbivory were characterised by the red algae *Amphiroa* sp. and *Amansia* sp., both of which are actively avoided by some siganids [57], [63]. These findings are consistent with other studies that show a restriction of palatable species to areas of the reef with low levels of herbivory [33], [64].

### Feedback mechanisms between herbivory and algae

In many terrestrial systems, herbivores have a positive effect on the plants they consume through enhancing nutrient recycling and availability, a feedback mechanism known as grazing optimisation [12], [13], [65]. However, in our cage experiments macroalgae that were exposed to herbivores had the lowest nitrogen concentrations. Our results are consistent with several marine studies that show a short-term reduction in nitrogen content in seagrasses grazed by fish and urchins [66], [67]. This lack of a fertilisation effect may be partly due to the fact that the localised input of nutrients that occurs in terrestrial systems via the faeces and urine of herbivores is likely to be reduced in the marine environment, generally due to the dilution and dispersion of nutrients via water movement. Indeed, the only marine examples where optimisation effects have been recorded through excretion of nitrogenous wastes of herbivores are from shallow, poorly flushed systems [68]. Other examples of grazing optimization effects in the marine environment come from systems dominated by specialist herbivores such as turtles, who generally raise the nitrogen content of seagrasses through increasing the proportion of nutrient-rich new foliage by repeated cropping [69], [70], [71]. Additionally, herbivores can also indirectly enhance the nitrogen content of macrophytes by inducing bacterial nitrogen fixation either through sediment disturbance (e.g. effects of dugongs on seagrass meadows; [71]) or by removing algal recruits and facilitating dominance by rapidly colonising nitrogen-fixing cyanobacteria [72], [73]. While the lack of nitrogen enhancement of transplanted algae in our cage experiment could be partly due to the short duration of the trial (6 weeks), the fact that we found no differences in the nitrogen content of *Lobophora variegata* specimens from habitats with contrasting levels of herbivory (lagoon and reef flat morphotypes) indicates that potential differences in nitrogen fixation or other nitrogen uptake mechanisms between habitats are not having an effect on macroalgal nitrogen content in Ningaloo Reef.

### Herbivory patterns explained by structural complexity

In this study, structural complexity was identified as the key feature mediating spatial patterns of macroalgal consumption by fish. This conclusion is supported by three lines of evidence: (1) different outcomes from experimental exclusion of herbivorous fish in structurally-complex reef habitat and structurally-simple lagoon habitat; (2) a strong linear relationship between macroalgal consumption and structural complexity; and, (3) a decrease in herbivory with increasing distance from structurally-complex reef habitat. These results are consistent with other studies that have shown an increase in herbivore density and grazing rates with topographical complexity [33], [49], [74], [75]. Although there is a lack of experimental studies identifying the specific causes that link herbivory and structure, availability of shelter or refuges, increased diversity of microhabitats and resource partitioning are thought to be key influences [76]. Complex habitats can reduce predation by providing shelter [77], lower competition through increased niche availability [78], [79], and provide specific settlement habitat for larvae [80]. Nevertheless, greater structural complexity is not associated with higher herbivory at all spatial scales. For example, herbivory is lower within branching coral habitats that are highly structured at small scales (cm) than in nearby planar coral habitats [62].

Understanding the mechanisms that drive spatial patterns of ecological processes in coral reefs is particularly important for the management of these systems, because the ability of individual coral reefs to reorganise and maintain ecosystem function following disturbance is considered to strongly depend on the matrix of adjacent reefs and habitats in the surrounding seascape [81], [82]. This study highlights the importance of structural complexity in establishing spatial patterns of macroalgal fish herbivory, an ecological process of key importance that can reverse phase shifts when algae overgrow corals following disturbances [47]. Structural complexity thus emerges as a critical feature of reefs that is essential for the healthy functioning of the ecosystem.

## Materials and Methods

The Department of the Environment and Conservation of Western Australia provided a permit to the authors during 2008–2009 to perform this study within the Sanctuary Zones of the Ningaloo Marine Park (Permit Number CE002084) and to collect flora samples (Permit Number SF006457).

### Study area

This study was conducted at Ningaloo Reef (Western Australia), a fringing reef approximately 290 km in length. Ningaloo Reef is an arid-zone reef where extensive coral reefs occurs in close proximity to the mainland. The study was conducted between April 2008 and September 2009 in the Mandu (22° 06′ S, 113° 52′ E) and Maud (23° 07′ S, 113° 44′ E) sanctuary zones of the Ningaloo Marine Park. At Ningaloo, the reef crest is narrow and mostly devoid of coral growth, the reef flat landward of the reef crest hosts coral communities across a width of approximately 150 m, and the outer reef slopes seaward of the reef crest presenting a well-developed spur and groove morphology to depths of 30 m [83] (Fig. S1). In the Mandu sanctuary zone, the reef encloses a lagoon that is about 1 km in width. In the Maud sanctuary zone, the width of the lagoon ranges more widely from 1.7 km to 7 km. In each location, the lagoon is populated with sparse corals, sandy substrata and patches of macroalgae. The tides in the area have a maximum ∼2 m range at spring tides.

Most of this study took place in the Mandu sanctuary zone, where we haphazardly selected three study sites in each of three habitats that characterise the area: lagoon, reef flat and outer reef (Fig. S1). We performed one experiment (on the effects of reef proximity on macroalgal removal) in the Maud sanctuary zone, where we haphazardly selected three study sites at the boundary between the reef flat and lagoon, with each site located about 250 m apart.

### Patterns in rates of consumption of macroalgae among habitats

Relative differences in consumption of erect macroalgae between lagoon, reef flat and outer reef habitats at all Mandu sites were measured in April 2008 using tethered pieces of *Sargassum myriocystum*. This brown alga is rapidly eaten (within hours) and has a relatively simple morphology, which allowed us to estimate biomass loss from differences in length before and after deployment. *S. myriocystum* plants bear 3–6 main lateral branches per individual, and each of these has a consistent length-weight relationship (148.01±9.241 mg/cm). Patterns of herbivory were determined by placing lateral branches of *S. myriocystum* of 15 cm in length and similar weight (mean 2.13±0.19 g; n = 26) in three sites at each of the lagoon, reef flat and outer reef habitats. In each site, *S. myriocystum* lateral branches (n = 25) were distributed haphazardly and tethered to the available substrata using cable ties. In the lagoon habitat, branches were either tethered to other macroalgae or to lose pieces of dead coral on the sand at around 1.5 m depth. In the reef flat habitat, branches were mostly tethered to pieces of dead coral covered in epilithic algal matrix adjacent to live corals, at 1–2 m depth. In the outer reef habitat, branches were mostly tethered to coral pieces and crustose coralline rocky surfaces at about 6 m depth – the shallowest depth that we could easily access in regular swell conditions. Tethered algae were collected 4–7 hours after deployment, and mass consumed was estimated from the total length consumed, and converted to mass consumed per hour. Some tethered algae became detached and lost, leading to an unbalanced data set (final n ranged from 17 to 25 depending on site). Replicates where algae became wholly detached were not included in the analysis because we could not be sure that detachment was due to herbivory.

### Patterns in biomass and species composition of herbivorous fish among habitats

Censuses of the herbivorous fish assemblage were carried out during a two-week period in November 2008 at three sites in each of the three habitats in the Mandu Sanctuary zone (Fig. 1). Fishes were counted along eight 25×5 m haphazardly placed transects per site during daylight hours (avoiding 2 hours after sunrise and before sunset). Fish counts were performed swimming at a constant speed (ca. 8 minutes per 25 m transect) and counting and estimating the size of fish within 2.5 m of either side of the transect line. Fishes were identified to species level and their total length was estimated in 5 cm size categories. Size estimates were validated using objects of known length. Length estimates for individual fish were converted to biomass using the allometric length-weight conversion W =  a * TL^b^, where W is weight in grams, TL is total length and parameters *a* and *b* are constants obtained from the literature [84]. We restricted our counts to mobile herbivorous and ‘nominally’ herbivorous fishes, excluding pomacentrids [85]. We identified 25 species from the families Acanthuridae, Siganidae, Kyphosidae and Labridae (parrotfishes). These data were analysed in two ways: one including all species of roving herbivorous fish and one including only species that are considered to be browsers (consumers of macroalgae). Of the species recorded, 11 taxa were identified as browsers of erect macroalgae based on gut content analyses [86] and direct observations on remote video cameras (unpubl. data): *Kyphosus vaigiensis*, *Naso lituratus*, *Naso* spp., *Naso unicornis*, *Scarus ghobban*, *S. schlegeli*, initial-phase *Scarus* sp., *Siganus argenteus*, *S. doliatus*, and *S. trispilos*.

### Patterns in cover, biomass and species composition of macroalgae among habitats

To determine whether spatial patterns in macroalgal herbivory were related to macroalgal distribution, we measured algal cover, algal biomass and community composition at each site at Mandu in November 2008. Algal cover was quantified using the line intercept benthic survey method described by Fox and Bellwood [30]. We conducted a total of 6 replicate transects (total of 30 points per replicate) in each of the habitats at the three sites.

Macroalgal biomass and community composition were measured by clearing three 0.25 m^2^ haphazardly placed quadrats of all macroalgae (arbitrarily defined as algae with thallus larger than 1 cm) at each site. Algal samples were bagged and returned to the laboratory, where they were sorted to genus level (where possible) and weighed. Algal taxa that we were unable to identify were classified according to broad functional groups (brown, green or red; filamentous, encrusting or foliose).

### Patterns in coral cover and structural complexity

To determine whether patterns in consumption of macroalgae were related to topographic complexity, we measured coral cover (which provides three-dimensional structure and potential refuges) and estimated a rugosity ratio (n = 3) at each site. Live coral cover was quantified using the line intercept benthic survey method described above for algal cover. To measure rugosity, a 10 m light chain was placed along the substrate contour, and the equivalent straight line horizontal distance encompassed by the 10 m of chain was measured (n = 3). The rugosity ratio (R) was calculated as the straight line horizontal distance along the reef divided by the total chain length, with values close to unity indicating a flat substratum and lower values indicating a structured habitat [87].

### Experimental test of effects of habitat and herbivory on algal consumption, productivity and chemical composition

A transplant experiment was set up to determine the effects of habitat on the consumption and growth of algae and to assess the interactive effects of habitat and herbivory on algal chemical traits. The experiment took place over 6 weeks from April to May 2008 in the Mandu sanctuary zone. Specimens of *Lobophora variegata* (ruffled morphotype, *sensu* Coen and Tanner [53]) were randomly collected from a lagoon location and placed on reef flat and lagoon habitats under caged and uncaged conditions. This species was chosen because it is commonly found in all coral-reef habitats and because preliminary feeding trials showed it was consumed at a lower rate than other macroalgae (unpubl. data), thus making it more suitable for long-term transplant experiments than other species that are consumed within hours when placed in the reef flat. Three *L. variegata* thalli were placed within each plot. All plots were randomly distributed and placed about 3–5 m apart from each other.

We used triangular cages of 1082 cm^2^ (equilateral triangle with sides of 50 cm and 50 cm in height). Open (uncaged) plots were marked with steel reinforcing bar along the corners. In caged plots, fences and roofs were made with plastic-coated metal mesh (2.5 cm mesh size), thereby excluding large herbivorous fish. Partial cages consisted of steel reinforcing bars along the corners with one fenced side and a roof, and were used to control for cage artefacts. All plots had a base of plastic coated mesh to which thalli were attached. The experiment ran for 6 weeks, and the cages were cleaned of fouling organisms once after 2 weeks, although these were not abundant. We recorded blotted wet-weight of algae (n = 3) at the beginning and at the end of the experiment and calculated biomass change.

At the end of the experiment, algal thalli were freeze-dried and ground. Nitrogen and carbon content of individual thalli were analysed using a Europa Scientific ANCA-NT 20-20 mass spectrometer. Where possible, *L. variegata* sections of intermediate age (i.e. equidistant from holdfast and thallus edge) were used in the chemical analyses, and care was taken to gently remove any epibiota. Some thalli were so heavily grazed that we used the entire thallus in the analysis, and in some instances we did not have enough mass to conduct chemical analyses, leading to an unbalanced data set.

### Experimental test of palatability of algae from high and low herbivory habitats

We compared the palatability and chemical composition of *Lobophora variegata* from high and low herbivory habitats (reef flat and lagoon, respectively) with an experiment performed in April 2008 in the Mandu sanctuary zone. Lagoon and reef flat habitats are characterised by hosting different morphotypes of *L. variegata*. As in other coral-reef ecosystems, an erect ruffled form (hereafter referred to as ‘lagoon’ morphotype) is usually found on sandy substrata where herbivores are less abundant, whereas the flat decumbent form (hereafter referred to as ‘reef flat’ morphotype) is usually found underneath coral plates in coral-dominated habitat where herbivores are often more common [53]. The lagoon and reef flat morphotypes of *L. variegata* were offered in pairs of similar initial area in the reef-flat habitat, where we recorded highest herbivore activity. Replicate pairs (n = 15) were at least 3 metres apart from each other. An equal number of controls (n = 15) were individually protected from herbivores with plastic window-screen mesh cages (3 mm mesh size). *L. variegata* pairs were left in the field for five days. Replicates with one or two wholly detached algae were not included in the analysis because we could not guarantee that detachment was due to herbivory. Photographs of each algal specimen were taken at the beginning and at the end of the experiment and consumption was measured as changes in area determined using ImageJ analysis software. Five additional thalli of each morphotype were collected at the beginning of the experiment for carbon, nitrogen and phenolic chemical analyses to further identify potential differences in nutritional traits between habitats. Total phenolic content was quantified spectrophotometrically using a modified Folin-Ciocalteu assay [88].

### Effect of proximity to reef on consumption of macroalgae

To test the effects of proximity to reef on macroalgal removal, we tethered *Sargassum myriocystum* lateral branches at a range of distances from the reef flat/lagoon boundary and measured the amount of algae consumed after 48 hours. We predicted that if structural complexity positively influences herbivory, the rates of macroalgal removal would be higher near the structurally complex reef flat habitat than in the flat lagoon habitat. This experiment was performed in the Maud sanctuary zone. We were unable to perform this experiment in the Mandu sanctuary zone because the reef flat/lagoon boundary in that part of the Ningaloo reef is diffuse, with isolated coral heads scattered irregularly near the boundary. In contrast, the reef flat and lagoon habitats are clearly defined at Maud. These two sanctuary zones have similar rates of macroalgal removal and a similar herbivorous fish assemblage (Michael et al., unpublished data). *Sargassum myriocystum* (n = 3) lateral branches of about 40 cm in length were tethered at 9 distances relative to the reef flat/lagoon boundary (−25, 0, 5, 10, 15, 25, 30, 50 and 75 m) at each of three separate sites. Replicates within each distance per site were approximately 15 m apart and parallel to the reef flat/lagoon boundary, and sites were approximately 250 m apart. The length of each individual *S. myriocystum* lateral branch was measured at the beginning and at the end of the experimental period. Since sand was the most common substrate away from the reef, *S. myriocystum* lateral branches were tethered to lose pieces of dead coral that were buried in the sand.

### Statistical analyses

All data were checked for normality and equality of variances by visual inspection of scatterplots and distribution of residuals [89]. Where appropriate, data were transformed to conform to parametric assumptions. When assumptions of normality could not be met, the significance of effects was assessed by permutation [90].

Patterns in consumption of tethered algae, total fish biomass, coral cover and in the species composition of herbivorous fish and algae communities were analysed using PERMANOVA testing for differences between sites (3 levels, random factor) and habitats (3 levels, fixed factor). Patterns in the cover and biomass of macroalgae and in rugosity were analysed using analysis of variance (ANOVA) with the same design. When significant differences were detected between main effects in ANOVA, Student-Newman-Keuls (SNK) tests were used to resolve the differences among means.

Bray-Curtis distance was our metric in all multivariate analyses and data were fourth-root transformed prior to analyses to reduce the effects of numerically large values (i.e. abundant schooling species) [91]. Multivariate differences of fish and algae communities between sites and habitats were visualised using CAP. This procedure produces a constrained ordination and presents data on the axes chosen to best distinguish groups in the data [92]. CAP also provides misclassification errors by carrying out a leave-one-out allocation of observations to groups (habitats), thus indicating the robustness of the classification. In addition, species with the highest contribution to differences among habitats were identified as those that had the highest absolute Pearson correlation with the canonical axis from the CAP analysis. A correlation of r >0.4 was used as an arbitrary cut-off to display potential relationships between individual species and the canonical axes.

We used regressions to determine whether rates of herbivory matched patterns in algal cover, algal biomass and rugosity, and to assess the relationship between herbivory rates and roving herbivorous and browser fish biomass. All data were averaged at the site level. Linear, polynomial and logarithmic regressions were fitted to the data, and the significant regression that best-fit the data was selected using Akaike's Information Criterion.

In the herbivore exclusion x habitat experiment, changes in biomass and chemical composition in *L. variegata* after 6 weeks were analysed using a four-way ANOVA, testing for differences among sites (3 levels, random), habitats (2 levels, fixed), large herbivore-exclusion treatments (termed ‘herbivory effect’ throughout; 3 levels, fixed) and plots (3 levels, random and nested in the interaction of Site x Habitat x Herbivory). Differences in feeding between lagoon and reef flat *L. variegata* were analysed using a t-test as outlined by Peterson and Renaud [93] to adequately incorporate controls for mass changes not due to consumption. The t-statistic was calculated by comparing the between-food differences in loss of mass of treatments (Choice 1 – Choice 2, with herbivores) with the between-food differences in loss of mass of control replicates (Choice 1 – Choice 2, without herbivores). Differences in macroalgal chemical traits between morphotypes were analysed using two-sample t-test when variances were homogenous or Welch's t-test otherwise. Confidence intervals (CI) at 95% of t-test results are presented to assess the validity of non-significant results following Colegrave and Ruxton [94]. The effects of proximity to reef on macroalgal removal were analysed using a two-way ANOVA testing for differences among sites (3 levels, random) and distances (9 levels, fixed).

All PERMANOVA and multivariate analyses were performed using Primer-E v6 software [95] with the PERMANOVA+ add-on package (version 1.0.1; [96]). All ANOVAs were performed using the statistical package GMAV5 (coded by A. J. Underwood and M. G. Chapman, University of Sydney, Australia). T-tests and regression analyses were performed using R software (Version 2.9.0) [97].

## Supporting Information

Figure S1 **Study area: Mandu and Maud sanctuary zones in Ningaloo Reef, Western Australia.** In Mandu, there were three sites within each habitat: lagoon (diamond icons), reef flat (circle icons) and outer reef (square icons).
(EPS)

Table S1 **PERMANOVA results on removal rates of** ***Sargassum myriocystum*** **lateral branches between sites and habitats.**
(DOCX)

Table S2 **Results of the two factor univariate analyses assessing differences in (a) all roving herbivore biomass, (b) macroalgal browser biomass, (c) algal cover, (d) total algal biomass, (e) coral cover and (f) rugosity between sites and habitats (a - b PERMANOVA; c – f ANOVA).** Pooling procedure was used in accordance to Underwood [98].
(DOCX)

Table S3 **Results of the two factor PERMANOVAs assessing multivariate differences between sites and habitats in assemblages of (a) all roving herbivorous fish, (b) browser herbivorous fish and (c) algae.**
(DOCX)

Table S4 **ANOVA results on (a) biomass change and PERMANOVA results on changes in (b) nitrogen and (c) C/N of** ***Lobophora variegata*** **transplanted specimens in lagoon and reef flat habitats over the three sites.**
(DOCX)

Table S5 **ANOVA results on removal of** ***Sargassum myriocystum*** **lateral branches at three sites and nine distances from the reef flat/lagoon boundary.**
(DOCX)
