# Supplementary material for: Spatial Patterns in Herbivory on a Coral Reef Are Influenced by Structural Complexity but Not by Algal Traits
Source: PLoS One. 2011 Feb 11;6(2):e17115. doi: 10.1371/journal.pone.0017115 (PMC3037963; doi:10.1371/journal.pone.0017115)
Supplement: Table S1 — PERMANOVA results on removal rates of Sargassum myriocystum lateral branches between sites and habitats. (DOCX) [file pone.0017115.s002.docx]

**Table S1.** Results of the permutational analysis of variance (PERMANOVA) assessing differences in removal rates of *Sargassum myriocystum* lateral branches between sites and habitats.

| Source of variation | df | MS | Pseudo-F | P |
| --- | --- | --- | --- | --- |
| Site (S) | 2 | 1.681 | 0.869 | 0.405 |
| Habitat (H) | 2 | 76.479 | 28.005 | **0.011** |
| S x H | 4 | 2.734 | 1.413 | 0.22 |
| Residual | 193 | 1.935 |  |  |

Relevant significant probabilities are indicated in bold.
