# Supplementary material for: Spatial Patterns in Herbivory on a Coral Reef Are Influenced by Structural Complexity but Not by Algal Traits
Source: PLoS One. 2011 Feb 11;6(2):e17115. doi: 10.1371/journal.pone.0017115 (PMC3037963; doi:10.1371/journal.pone.0017115)
Supplement: Table S2 — Results of the two factor univariate analyses assessing differences in (a) all roving herbivore biomass, (b) macroalgal browser biomass, (c) algal cover, (d) total algal biomass, (e) coral cover and (f) rugosity between sites and habitats (a - b PERMANOVA; c – f ANOVA). Pooling procedure was used in accordance to Underwood [98]. (DOCX) [file pone.0017115.s003.docx]

**Table S2.** Results of the two factor univariate analyses of variance assessing differences between sites and habitats in (a) all roving herbivore biomass, (b) macroalgal browser biomass, (c) algal cover, (d) total algal biomass, (e) coral cover and (f) rugosity (a - b PERMANOVA; c – f ANOVA).

|  | (a) All roving herbivores | | | |  | (b) Macroalgal browsers | | | | |  | (c) Algae cover | | | |
| --- | --- | --- | --- | --- | --- | --- | --- | --- | --- | --- | --- | --- | --- | --- | --- |
| Source of variation | df | MS | Pseudo-F | P |  | df | MS | Pseudo-F | | P |  | df | MS | F | P |
| Site (S) | 2 | 3.74 x10^8^ | 1.93* | 0.116 |  | 2 | 2.42 x10^6^ | 0.551† | | 0.598 |  | 2 | 1.463 | 9.41 | **< 0.001** |
| Habitat (H) | 2 | 1.03 x10^9^ | 5.30* | **0.003** |  | 2 | 2.95 x10^7^ | 6.701† | | **0.004** |  | 2 | 4.612 | 13.42 | **0.017** |
| S x H | 4 | 1.86 x10^8^ | 0.96 | 0.464 |  | 4 | 5.84 x10^6^ | 1.357 | | 0.264 |  | 4 | 0.344 | 2.21 | 0.083 |
| Residual | 63 | 1.94 x10^8^ |  |  |  | 63 | 4.31 x10^6^ |  | |  |  | 45 | 0.1554 |  |  |
|  | (d) Algae biomass | | | |  | (e) Coral cover | | | | |  | (f) Rugosity | | | |
| Source of variation | df | MS | F | P |  | df | MS | | F | P |  | df | MS | F | P |
| Site (S) | 2 | 4.216 | 2.11‡ | 0.145 |  | 2 | 474.07 | | 5.512 | **0.008** |  | 2 | 0.010 | 0.50 | 0.617 |
| Habitat (H) | 2 | 13.803 | 6.91‡ | **0.005** |  | 2 | 8155.6 | | 21.327 | **0.028** |  | 2 | 0.876 | 25.52 | **0.005** |
| S x H | 4 | 1.392 | 0.65 | 0.633 |  | 4 | 382.41 | | 4.446 | **0.005** |  | 4 | 0.034 | 1.74 | 0.185 |
| Residual | 18 | 2.133 |  |  |  | 18 | 3870.4 | |  |  |  | 18 | 0.020 |  |  |

Significant probabilities are indicated in bold. Pooling procedure was used in accordance to Underwood [98]

Tested against the pooled term = S x H + Residual: * all roving herbivorous fish analysis MS = 1.93 x 108, df = 67; † browser fish analysis MS = 4.34 x 106, df = 67; ‡ algae analysis MS = 1.99, df = 22
