# Supplementary material for: Spatial Patterns in Herbivory on a Coral Reef Are Influenced by Structural Complexity but Not by Algal Traits
Source: PLoS One. 2011 Feb 11;6(2):e17115. doi: 10.1371/journal.pone.0017115 (PMC3037963; doi:10.1371/journal.pone.0017115)
Supplement: Table S3 — Results of the two factor PERMANOVAs assessing multivariate differences between sites and habitats in assemblages of (a) all roving herbivorous fish, (b) browser herbivorous fish and (c) algae. (DOCX) [file pone.0017115.s004.docx]

**Table S3.** Results of the two factor PERMANOVAs assessing multivariate differences between sites and habitats in assemblages of (a) all roving herbivorous fish, (b) browser herbivorous fish and (c) algae.

|  | | | | | | | | | | | | | | |
| --- | --- | --- | --- | --- | --- | --- | --- | --- | --- | --- | --- | --- | --- | --- |
|  | (a) All roving herbivores | | | |  | (b) Macroalgal browsers | | | |  | (c) Algae | | | |
| Source of variation | df | MS | Pseudo-F | P |  | df | MS | Pseudo-F | P |  | df | MS | Pseudo-F | P |
| Site (S) | 2 | 5895.1 | 4.6235 | **0.001** |  | 2 | 2416.2 | 1.5877 | 0.116 |  | 2 | 1832.8 | 1.6993 | 0.089 |
| Habitat (H) | 2 | 40310 | 6.5499 | **0.005** |  | 2 | 15085 | 2.9007 | **0.034** |  | 2 | 5446 | 2.340 | 0.051 |
| S x H | 4 | 6154.3 | 4.8268 | **0.001** |  | 4 | 5200.5 | 3.4173 | **0.001** |  | 4 | 2327.2 | 2.1576 | **0.006** |
| Residual | 63 | 1275 |  |  |  | 63 | 1521.8 |  |  |  | 18 | 1078.6 |  |  |

All data were fourth-root transformed prior to analyses. Significant probabilities are indicated in bold.
