# Supplementary material for: Spatial Patterns in Herbivory on a Coral Reef Are Influenced by Structural Complexity but Not by Algal Traits
Source: PLoS One. 2011 Feb 11;6(2):e17115. doi: 10.1371/journal.pone.0017115 (PMC3037963; doi:10.1371/journal.pone.0017115)
Supplement: Table S4 — ANOVA results on (a) biomass change and PERMANOVA results on changes in (b) nitrogen and (c) C/N of Lobophora variegata transplanted specimens in lagoon and reef flat habitats over the three sites. (DOCX) [file pone.0017115.s005.docx]

**Table S4.** ANOVA results on (a) biomass change and PERMANOVA results on changes in (b) nitrogen and (c) C/N of *Lobophora variegata* transplanted specimens in lagoon and reef flat habitats over the three sites and three herbivory treatments (open, caged and partially caged).

|  | (a) Biomass change | | | |  |  | (b) Nitrogen | | |  | (c) Carbon/ Nitrogen | | |
| --- | --- | --- | --- | --- | --- | --- | --- | --- | --- | --- | --- | --- | --- |
| Source of variation | df | MS | F | P |  | df | MS | Pseudo F | P |  | MS | Pseudo F | P |
| Site (S)* | 2 | 21.457 | 0.44 | 0.646 |  | 2 | 4.273 x10^-2^ | 1.44 | 0.267 |  | 1.600 | 0.143 | 0.873 |
| Habitat (H) | 1 | 884.615 | 22.81 | **0.0412** |  | 1 | 0.147 | 6.834 | 0.107 |  | 300.94 | 96.42 | **0.007** |
| Herbivory (He) | 2 | 280.023 | 7.87 | **0.0411** |  | 2 | 0.256 | 12.03 | **0.016** |  | 24.591 | 2.609 | 0.18 |
| Plot (SxHxHe) | 36 | 48.040 | 2.89 | **< 0.001** |  | 32 | 3.078 x10^-2^ | 1.827 | **0.019** |  | 11.619 | 1.815 | **0.017** |
| S x H | 2 | 38.780 | 0.8* | 0.457 |  | 2 | 2.139 x10^-2^ | 0.721 | 0.453 |  | 2.985 | 0.77 | 0.77 |
| S x He | 4 | 35.576 | 0.73* | 0.575 |  | 4 | 2.117 x10^-2^ | 0.709 | 0.597 |  | 9.450 | 0.834 | 0.505 |
| H x He | 2 | 277.880 | 5.73* | **0.007** |  | 2 | 5.459 x10^-2^ | 0.839 | 0.492 |  | 21.839 | 1.597 | 0.311 |
| S x H x He | 4 | 52.648 | 1.1 | 0.377 |  | 4 | 6.59 x10^-2^ | 2.206 | 0.099 |  | 13.751 | 1.219 | 0.323 |
| Residual | 108 | 16.648 |  |  |  | 92 | 1.684 x10^-2^ |  |  |  | 6.400 |  |  |
| Residual | 63 | 1.94 x10^8^ |  |  |  | 63 | 4.31 x10^6^ |  |  |  | 2.133 |  |  |

Pooling procedure was used in accordance to Underwood [98]. Relevant significant probabilities are indicated in bold.

* Tested against the pooled term = S x H x He + Plot (SxHxHe); MS = 48.5, df = 40
