# Supplementary material for: Spatial Patterns in Herbivory on a Coral Reef Are Influenced by Structural Complexity but Not by Algal Traits
Source: PLoS One. 2011 Feb 11;6(2):e17115. doi: 10.1371/journal.pone.0017115 (PMC3037963; doi:10.1371/journal.pone.0017115)
Supplement: Table S5 — ANOVA results on removal of Sargassum myriocystum lateral branches at three sites and nine distances from the reef flat/lagoon boundary. (DOCX) [file pone.0017115.s006.docx]

**Table S5.** ANOVA results on removal of *Sargassum myriocystum* lateral branches at three sites and nine distances from the reef flat/ lagoon boundary.

| Source of variation | df | MS | F | P |
| --- | --- | --- | --- | --- |
| Site (S) | 2 | 1084.114 | 15.63 | **<0.001** |
| Distance (D) | 8 | 1561.181 | 6.91 | **<0.001** |
| S x D | 16 | 225.781 | 3.26 | **<0.001** |
| Residual | 54 | 69.364 |  |  |

Significant probabilities are indicated in bold.
